# Supplementary figures and images for: A bipartite, low-affinity roadblock domain-containing GAP complex regulates bacterial front-rear polarity
Source: PLoS Genet. 2022 Sep 6;18(9):e1010384. doi: 10.1371/journal.pgen.1010384 (PMC9481161; doi:10.1371/journal.pgen.1010384)

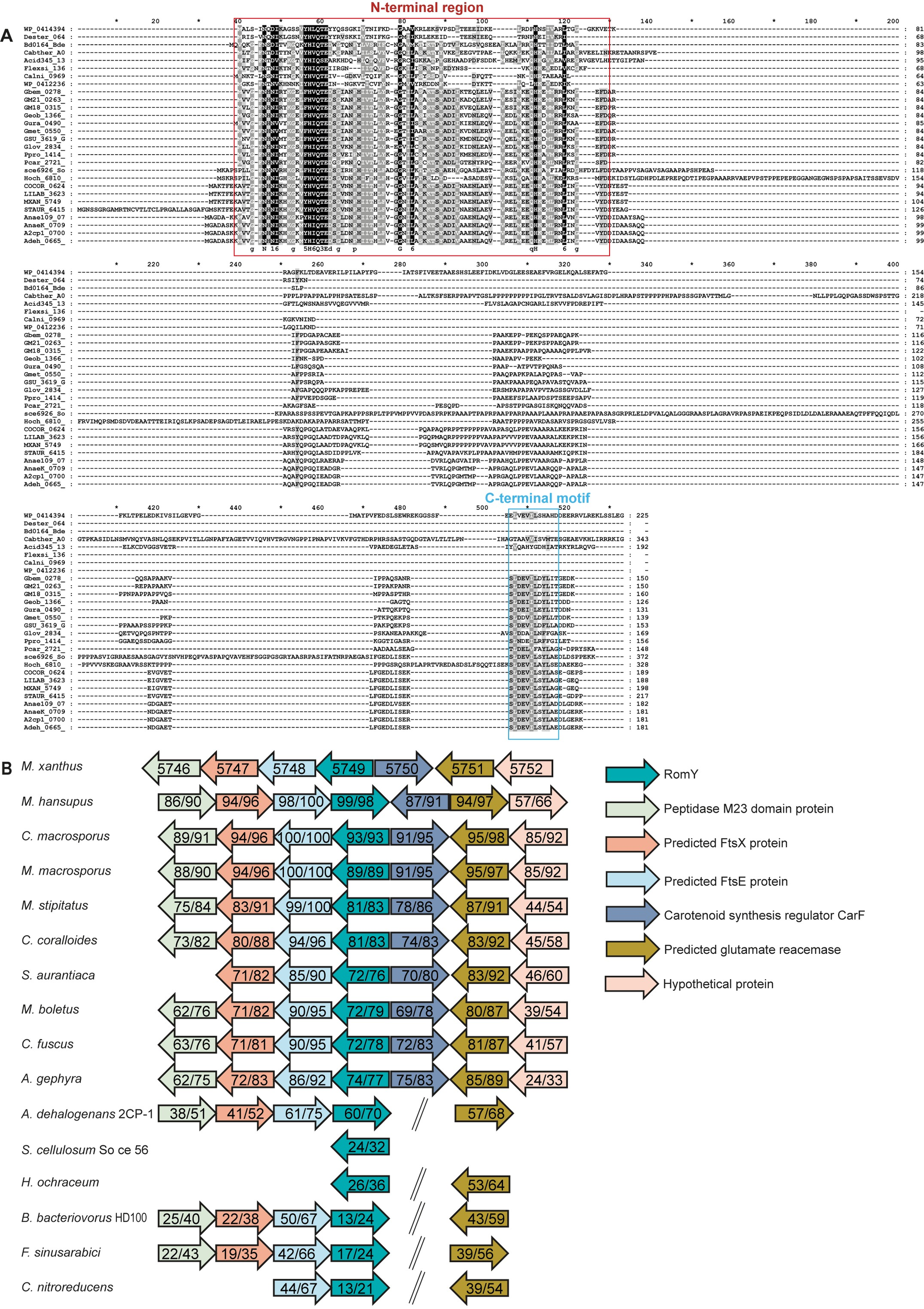

Supplement: S1 Fig — A. Sequence alignment of RomY homologs. In red, the conserved N-terminal region, and light blue, the partially conserved C-terminal motif. B. The romY locus is partially conserved. Transcription direction is indicated by the orientation of arrows with MXAN numbers indicated for the romY locus in M. xanthus. Note that in the NCBI Reference Sequence NC_008095.1, MXAN_5746 to MXAN_5752 are reannotated as MXAN_RS27850 to MXAN_RS27880; % similarity/identity between homologs from M. xanthus and other species is indicated by numbers in the arrows. For the proteins encoded by genes flanking romY in M. xanthus, domains were identified using SMART [70]. % similarity/identity between protein homologs were calculated using EMBOSS Needle software (pairwise sequence alignment). All listed species belong to the order Myxococcales except for Bdellovibrio bacteriovorus HD100 that belongs to the class Oligoflexia and Flexistipes sinusarabica and Calditerrivibrio nitroreducens that belong to the class Deferribacteres. (TIF) [file pgen.1010384.s001.tif]

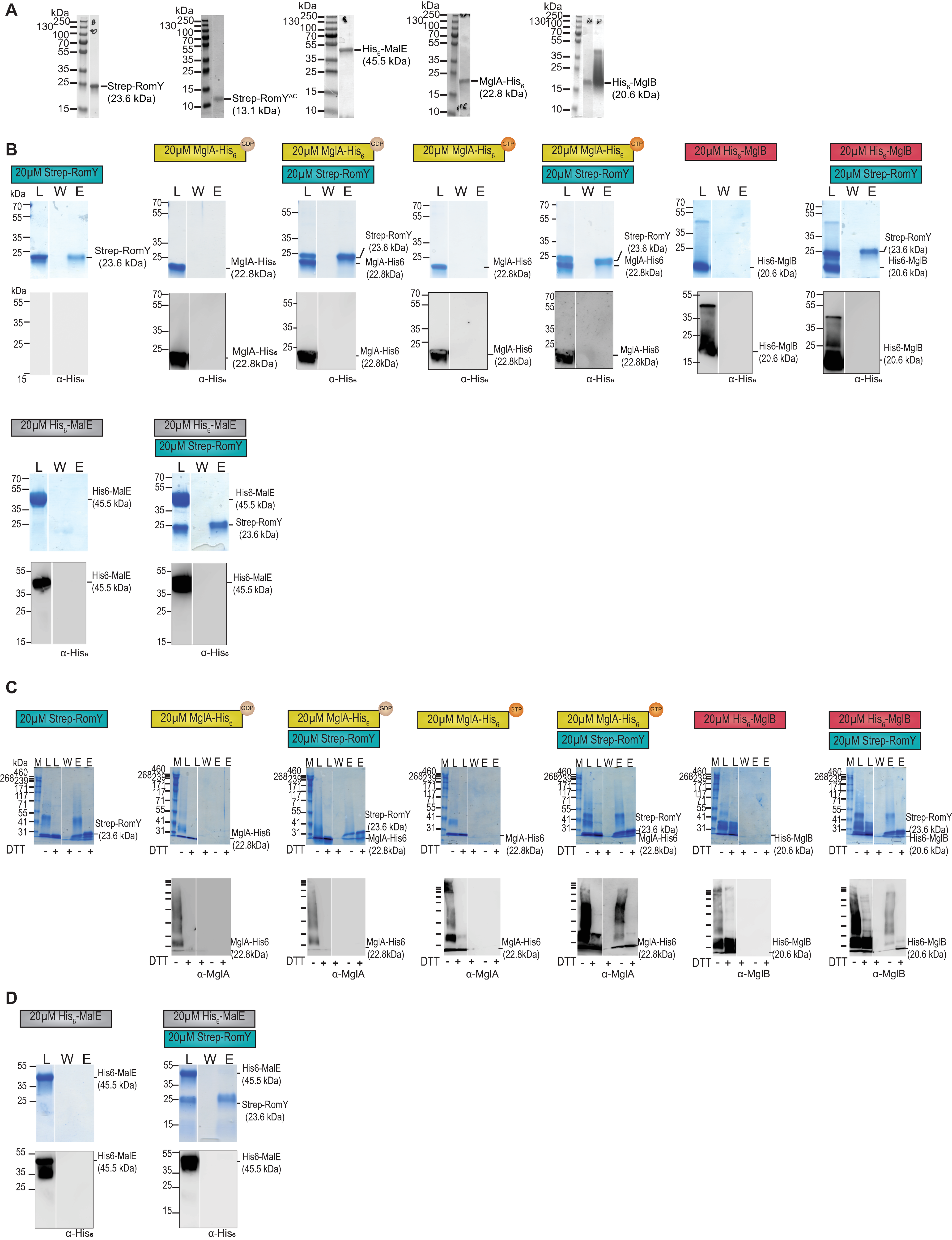

Supplement: S3 Fig — A. SDS-PAGE analysis of purified proteins used in in vitro assays. ~5-20ng of the indicated purified proteins were separated by SDS-PAGE and gels stained with Coomassie Brilliant Blue. Calculated molecular weight of the different proteins is indicated. Molecular size markers are indicated on the left. B. RomY interaction with MglA-GTP and MglB is not detected in the absence of DSP cross-linking. Proteins were mixed with final concentrations and 10mM GTP/GDP as indicated in the schematics for 30min at RT, and proteins applied to Strep-Tactin coated magnetic beads. Fractions before loading (L), the last wash (W) and after elution (E) were separated by SDS–PAGE, gels stained with Coomassie Brilliant Blue (upper panels) and subsequently probed with α-His6 antibodies (lower panels). All samples were prepared with loading dye supplemented with 100mM DTT. For each combination, fractions were separated on the same gel. Gaps between lanes indicate lanes deleted for presentation purposes. C. RomY interacts with MglA-GTP and MglB. Proteins were mixed with final concentrations and 10mM GTP/GDP as indicated in the schematics for 30min at RT, DSP added (final concentration 200μM, 5min, RT), DSP quenched, and proteins applied to Strep-Tactin coated magnetic beads. Fractions before loading (L), the last wash (W) and after elution (E) were separated by SDS–PAGE, gels stained with Coomassie Brilliant Blue (upper panels) and subsequently probed with α-MglA or α-MglB antibodies (lower panels). Eluted samples were treated with loading buffer with (+) or without (-) 100 mM DTT to break protein cross-links as indicated under the gels and immunoblots. For each combination, fractions were separated on the same gel. Gaps between lanes indicate lanes deleted for presentation purposes. Note that the experiments in Fig 2C are similar to those presented in this panel in which proteins were separated on a 7.5% acrylamide gel; proteins were separated on an 8–16% acrylamide gradient gel in the expe [file pgen.1010384.s003.tif]
