# Supplementary material for: A bipartite, low-affinity roadblock domain-containing GAP complex regulates bacterial front-rear polarity
Source: PLoS Genet. 2022 Sep 6;18(9):e1010384. doi: 10.1371/journal.pgen.1010384 (PMC9481161; doi:10.1371/journal.pgen.1010384)
Supplement: S2 Table — (DOCX) [file pgen.1010384.s009.docx]

| **S2 Table. *M. xanthus* strains used in this work** | | |
| --- | --- | --- |
| **Strain** | **Genotype** | **Source or reference** |
| DK1622 | Wild type | [1] |
| DK10410 | Δ*pilA* | [2] |
| SA5293 | Δ*aglQ* | [3] |
| SA5958 | Δ*romY* | This work |
| SA6920 | Δ*romY* P_nat__*romY* (*attB::*pDSZ36) | This work |
| SA4420 | Δ*mglA* | [4] |
| SA3387 | Δ*mglB* | [5] |
| SA3300 | Δ*romR* | [6] |
| SA3683 | Δ*romX* | [7] |
| SA3626 | Δ*mglA* Δ*romY* | This work |
| SA3630 | Δ*mglB* Δ*romY* | This work |
| SA3621 | Δ*romR* Δ*romY* | This work |
| SA5792 | Δ*romX* Δ*romY* | This work |
| SA3936 | Δ*mglB* Δ*romR* | [6] |
| SA3615 | Δ*mglB* Δ*romX* | [7] |
| SA8802 | Δ*frzE* | [7] |
| SA8193 | Δ*frzE* Δ*mglB* | This work |
| SA8316 | Δ*frzE* Δ*romY* | This work |
| SA9138 | *romY*^N^ | This work |
| SA9113 | Δ*mglB* P_van__*mglB* (*mxan18-19*::pDSZ31) | This work |
| SA9115 | Δ*mglB* Δ*romY* P_van__*mglB* (*mxan18-19*::pDSZ31) | This work |
| SA9114 | Δ*romY* P_van__*romY* (*mxan18-19*::pDSZ30) | This work |
| SA9117 | Δ*mglB* Δ*romY* P_van__*romY* (*mxan18-19*::pDSZ30) | This work |
| SA6901 | Δ*romY* P_nat__*romY-YFP* (*attB::*pDK132) | This work |
| SA6913 | Δ*mglA* Δ*romY* P_nat__*romY-YFP* (*attB::*pDK132) | This work |
| SA6903 | Δ*mglB* Δ*romY* P_nat__*romY-YFP* (*attB::*pDK132) | This work |
| SA6908 | Δ*romR* Δ*romY* P_nat__*romY-YFP* (*attB::*pDK132) | This work |
| SA8185 | *mglA-mVenus* | [7] |
| SA7577 | Δ*romY mglA-mVenus* | This work |
| SA7195 | *sgmX-mVenus* | [8] |
| SA11049 | Δ*romY sgmX-mVenus* | This work |
| SA3377 | *aglZ::aglZ-yfp* (pSL65) | [5] |
| SA9102 | Δ*romY aglZ::aglZ-yfp* (pSL65) | This work |
| SA10043 | *mglB-mVenus* | This work |
| SA10040 | Δ*romY mglB-mVenus* | This work |

**References**

1. Kaiser D. Social gliding is correlated with the presence of pili in *Myxococcus xanthus*. Proc Natl Acad Sci USA. 1979; 76:5952-6.

2. Wu SS, Kaiser D. Markerless deletions of *pil* genes in *Myxococcus xanthus* generated by counterselection with the *Bacillus subtilis sacB* gene. J Bacteriol. 1996; 178:5817-21.

3. Jakobczak B, Keilberg D, Wuichet K, Søgaard-Andersen L. Contact- and protein transfer-dependent stimulation of assembly of the gliding motility machinery in *Myxococcus xanthus*. PLOS Genet. 2015; 11:e1005341.

4. Miertzschke M, Körner C, Vetter IR, Keilberg D, Hot E, Leonardy S, et al. Structural analysis of the Ras-like G protein MglA and its cognate GAP MglB and implications for bacterial polarity. EMBO J. 2011; 30:4185-97.

5. Leonardy S, Miertzschke M, Bulyha I, Sperling E, Wittinghofer A, Søgaard-Andersen L. Regulation of dynamic polarity switching in bacteria by a Ras-like G-protein and its cognate GAP. EMBO J. 2010; 29:2276-89.

6. Keilberg D, Wuichet K, Drescher F, Søgaard-Andersen L. A response regulator interfaces between the Frz chemosensory system and the MglA/MglB GTPase/GAP module to regulate polarity in *Myxococcus xanthus*. PLOS Genet. 2012; 8:e1002951.

7. Szadkowski D, Harms A, Carreira LAM, Wigbers M, Potapova A, Wuichet K, et al. Spatial control of the GTPase MglA by localized RomR/RomX GEF and MglB GAP activities enables *Myxococcus xanthus* motility. Nat Microbiol. 2019; 4:1344-55.

8. Potapova A, Carreira LAM, Søgaard-Andersen L. The small GTPase MglA together with the TPR domain protein SgmX stimulates type IV pili formation in *M. xanthus*. Proc Natl Acad Sci USA. 2020; 117:23859-68.
