# Supplementary material for: A bipartite, low-affinity roadblock domain-containing GAP complex regulates bacterial front-rear polarity
Source: PLoS Genet. 2022 Sep 6;18(9):e1010384. doi: 10.1371/journal.pgen.1010384 (PMC9481161; doi:10.1371/journal.pgen.1010384)
Supplement: S3 Table — (DOCX) [file pgen.1010384.s010.docx]

**S3 Table. Plasmids used in this work**

| **Plasmid** | **Description** | **Reference** |
| --- | --- | --- |
| pSW105 | P*_pilA_*, *attP*, Kan^R^ | [1] |
| pSWU30 | Tet^R^, *attP* | [2] |
| pBJ114 | Kan^R^, *galK¸* vector for generating in-frame deletions | [3] |
| pMR3691 | *vanR* P_van_, Tet^R^ | [4] |
| pASK-IBA15+ | Vector for overexpression of Strep-tagged proteins; Kan^R^ | IBA Lifesciences GmbH |
| pET45b+ | Vector for overexpression of His_6_-tagged proteins; Ap^R^ | Merck Millipore |
| pDK95 | pBJ114; for generation of in-frame deletion of *romY* | This work |
| pDSZ36 | pSWU30; P_nat__*romY* | This work |
| pES2 | pBJ114; for generation of in-frame deletion of *mglB* | [5] |
| pDSZ35 | pBJ114; for generation of *romY^N^* | This work |
| pDSZ31 | pMR3691, *mglB*, Tet^R^ | This work |
| pDSZ30 | pMR3691, *romY*, Tet^R^ | This work |
| pDK132 | pSW105; P_nat__*romY-yfp*, Kan^R^ | This work |
| pLC20 | pBJ114; for *mglA* replacement by *mglA-mVenus* at native site | [6] |
| pAP35 | pBJ114; for *sgmX* replacement by *sgmX-mVenus* at native site | [7] |
| pSL65 | pBJ114; in-frame integration of *aglZ-gfp* at native site; Kan^R^ | [5] |
| pLC58 | pBJ114; for *mglB* replacement by *mglB-mVenus* at native site | This work |
| pTM1 | Overexpression MglA-His_6_ | [8] |
| pTM2 | Overexpression His_6_-MglB | [8] |
| pDSZ32 | Overexpression Strep-RomY | This work |
| pDSZ34 | Overexpression Strep-RomY^N^ | This work |
| pMAL-c6T | Overexpression His_6_-MalE | NEB |

**References**

1. Jakovljevic V, Leonardy S, Hoppert M, Søgaard-Andersen L. PilB and PilT are ATPases acting antagonistically in type IV pili function in *Myxococcus xanthus*. J Bacteriol. 2008; 190:2411-21.

2. Wu S, Kaiser D. Regulation of expression of the *pilA* gene in *Myxococcus xanthus*. J Bacteriol. 1997; 179:7748-58.

3. Julien B, Kaiser AD, Garza A. Spatial control of cell differentiation in *Myxococcus xanthus*. Proc Natl Acad Sci USA. 2000; 97:9098-103.

4. Iniesta AA, García-Heras F, Abellón-Ruiz J, Gallego-García A, Elías-Arnanz M. Two systems for conditional gene expression in *Myxococcus xanthus* inducible by isopropyl-thiogalactopyranoside or vanillate. J Bacteriol. 2012; 194:5875-85.

5. Leonardy S, Miertzschke M, Bulyha I, Sperling E, Wittinghofer A, Søgaard-Andersen L. Regulation of dynamic polarity switching in bacteria by a Ras-like G-protein and its cognate GAP. EMBO J. 2010; 29:2276-89.

6. Szadkowski D, Harms A, Carreira LAM, Wigbers M, Potapova A, Wuichet K, et al. Spatial control of the GTPase MglA by localized RomR/RomX GEF and MglB GAP activities enables *Myxococcus xanthus* motility. Nat Microbiol. 2019; 4:1344-55.

7. Potapova A, Carreira LAM, Søgaard-Andersen L. The small GTPase MglA together with the TPR domain protein SgmX stimulates type IV pili formation in *M. xanthus*. Proc Natl Acad Sci USA. 2020; 117:23859-68.

8. Zhang Y, Franco M, Ducret A, Mignot T. A bacterial Ras-like small GTP-binding protein and its cognate GAP establish a dynamic spatial polarity axis to control directed motility. PLOS Biol. 2010; 8:e1000430.
