# Supplementary material for: A bipartite, low-affinity roadblock domain-containing GAP complex regulates bacterial front-rear polarity
Source: PLoS Genet. 2022 Sep 6;18(9):e1010384. doi: 10.1371/journal.pgen.1010384 (PMC9481161; doi:10.1371/journal.pgen.1010384)
Supplement: Attachment — Submitted filename: Szadkowski_Rebuttal.docx [file pgen.1010384.s012.docx]

**Reviewer #1**:
The determinants of front-rear polarity in M. xanthus motility have been extensively studied over the past years. However it remained unknown how the GAP or GEF activity on MglA (the output of the polarity module) is dominant at the lagging or leading cell pole, respectively, while both the GEF and the GAP proteins (or protein complex in the case of the GEF) displayed the same asymmetric distribution at both poles. The authors investigated how the GEF and GAP activities are spatially separated and describe the protein RomY as weak interaction partner of MglB and a stimulator of its GAP function (“co-GAP”). The asymmetric localisation of RomY supports its co-GAP role being restricted at the lagging cell pole. While MglB is important for the recruitment of RomY, additional, unknown determinants of RomY localisation still remain to be discovered.
Overall this is a carefully conducted study, which combines bacterial genetics, microscopy, motility assays, enzymatic assays, revealing a new piece in the puzzle of Myxococcus polarity factors. Future research will be needed to decipher how the co-GAP function of RomY is achieved. The manuscript is well written and easy to read. I only have minor comments.
**Response:** Thank you very much for the very positive feedback!

Figure 1B: only one scale bar is shown while the legend indicates scale bars for left, middle and right.
**Response:** Thanks for pointing this out to us; in the revised figure, we included the missing scale bars.

Figure 2A: did the authors test whether the Strep-tagged RomY (used in in vitro assay to conclude on its GAP stimulating activity) complements the ∆romY motility defects similarly as the untagged version?
**Response:** We have not tested the Strep-RomY protein *in vivo*. However, we decided to use this construct because that allows us to compare the activity in vitro of full-length Strep-RomY to that of Strep-RomY^N^ (the variant that only contains the N-terminal domain of RomY). We would also like to mention that all the in vitro data obtained with this fusion are in agreement with the in vivo data obtained with RomY and RomY-YFP. We also have unpublished data demonstrating that a His6-RomY protein “behaves” like Strep-RomY in vitro.

Figure 2B: to support the interaction, it would have been nice to migrate side-by-side the crosslinked samples before and after reduction with DTT. The authors only show the post-DTT samples, while pre-DTT migration would have allowed to detect a band at higher MW corresponding to the expected size of the complex.
**Response:** Thanks for this suggestion. In the revised manuscript, we have rewritten this part of the Results section (line 237-248) and included new experiments in Fig. S3C with all the samples before and after DTT treatment to break the protein crosslinks. In this new experiment we probed the fractions with α-MglA and α-MglB antibodies rather than His6-antibodies to double check that the detected bands are MglA-His6 and His6-MglB interacting with Strep- RomY. And indeed, once again, we find that Strep-RomY interacts with MglA-His6 and His6-MglB.

Figure 5C: providing the complementation experiment would support the claim (role of RomY on MglA polar localisation)
**Response:** In Fig. 1B, we show that ectopically expressed *romY* complements the motility defects caused by the Δ*romY* mutation. Therefore, we decided not to include the complementation experiment again in Fig. 5C.

Figure 5D: it is unclear which strain/genotype is used here.
**Response:** Many apologies. We have now clarified the genotype in the figure.

At several places (e.g. lines 425, 426), the authors claim that MglB GAP activity is only stimulated at the lagging pole. Even though RomY is more prominently localised at the lagging pole, it can’t be excluded that the little fraction present at the leading pole could still turn on MglB GAP activity there as well (though to a lower extend, according to the weak interaction data). I would rather think that there is an overall dominance of the GEF activity over the GAP activity on the population of MglA proteins at the leading pole (and the opposite at the lagging pole), more than an exclusive GAP or GEF activity at either pole.
**Response:** Thank you for pointing this out to us. In the revised manuscript, we have rewritten the text throughout to emphasize that RomY by almost exclusively localizing at the lagging pole in moving cells contributes to stimulating MglB GAP activity at this pole and is essential for the MglB/RomY GAP activity to outcompete and dominate over the RomR/RomX GEF activity at this pole. Similarly, we emphasize that the RomR/RomX GEF complex dominate over the MglB(/RomY) GAP activity at the leading pole because there is little to no RomY at this pole. We also extended the Discussion to more clearly bring this idea across.

Text:
- Rephrase title of S7 Fig.
**Response:** Thanks & done.

- Line 454: adds instead of add.
**Response:** Thanks & done.

- S3 Fig. Legend: cross instead of crosss; same at line 977
**Response:** Thanks & done.

**Reviewer #2:**
The manuscript by Szadkowski et. al. describes the identification of a new component, RomY, in the front-rear polarity establishment and reversals in Myxococcus xanthus. They term RomY as a ‘co-GAP’ that activates the GAP action by MglB for stimulating the GTPase activity by MglA further. The authors present detailed in-vivo and some preliminary in-vitro experiments to validate their model of polarity regulation. Some of the claims regarding competition and low affinities of complex formation would require a finer set of experiments involving GTPase measurements at various stoichiometries of the interacting partners. The claim that RomRX GEF activity is outcompeted, again, remains a speculation unless proved by competition assays involving RomRX vs RomY.
**Response:** Thank you very much for the constructive feedback. In the revised manuscript (and as described in more details below), we followed the advice of the reviewer and included new experiments in which we measure MglA GTPase activity at various stoichiometries of MglA, MglB and RomY.

Concerning the statement that “*The claim that RomRX GEF activity is outcompeted, again, remains a speculation unless proved by competition assays involving RomRX vs RomY*”: The epistasis experiments in Fig 1D, E and Fig 4A, B do not support that RomY alone would be able to outcompete RomR/RomX GEF activity. They do also not support that RomY would inhibit RomR/RomX GEF activity. Rather they support that the bipartite MglB/RomY GAP outcompetes RomR/RomX activity at the lagging pole. Similarly, the *in vitro* experiments in Fig 2A and Fig 4C clearly show that RomY alone does not have GAP activity; rather the MglB/RomY complex has GAP activity and RomY stimulates MglB GAP activity. Because the idea that RomY alone would outcompete RomR/RomX GEF activity is not supported by any of our observations, we have not pursued this line of experimentation in vitro. We would like to add that we have also not done competition experiments in vitro with the RomR/RomX GEF and the MglB/RomY GAP because we do not know what the readout of this competition assay would be.

To clarify all these points, we have done are three things:
(1) we have rewritten the text throughout to emphasize that RomY by almost exclusively localizing at the lagging pole in moving cells contributes to stimulating MglB GAP activity at this pole and is essential for the MglB/RomY GAP activity to outcompete and dominate over the RomR/RomX GEF activity at this pole. We also emphasize that we have no evidence supporting that MglB/RomY interferes with RomR/RomX GEF activity. Similarly, we emphasize that the RomR/RomX GEF complex dominate over the MglB(/RomY) GAP activity at the leading pole because there is little to no RomY at this pole. We extended the Discussion to more clearly bring across these ideas. We would also like to add that all our data are in agreement with the competition model.
(2) Included experiments in which we titrate MglA with increasing concentrations of MglB and RomY in vitro. These experiments confirmed that RomY stimulates MglB GAP activity in vitro.
(3) Included additional pull-down experiments with cross-linking. These experiments also confirm that RomY interacts with MglA-GTP and MglB after cross-linking, thus supporting that the affinities of RomY for MglA-GTP and MglB are low.

Please see below some major and minor clarifications, to be sorted for improvement of the manuscript.

Major points:
1. For RomY to function as a coGAP, it appears that there is no stoichiometric requirement, based on the claim that overexpression of only one of the proteins compared to the other affected the activities, as observed in vivo. How can this be explained mechanistically?
**Response:** Because overproduction of MglB (in the presence of WT levels of RomY) and overproduction of RomY (in the presence of WT levels of MglB) results in increased GAP activity, we infer that in WT cells, RomY is not saturated by MglB and MglB is not saturated by RomY. MglB and RomY interact to form a complex and follow the general reaction scheme MglB2 + RomY ⇌ MglB2/RomY. Under steady state conditions, the ratio between MglB2 and RomY vs MglB2/RomY is constant. Thus, if MglB or RomY are overproduced in otherwise WT cells, this reaction is “pushed” towards formation of the MglB2/RomY complex. In other words, more of the MglB2/RomY complex will be generated independently of the precise stoichiometry of the MglB/RomY complex when MglB or RomY is overproduced. Consequently, the cellular level of the active MglB2/RomY GAP increases upon MglB or RomY overproduction.
The MglA/MglB complex has a stoichiometry of 1:2; however, we do not know the stoichiometry of the MglA/MglB/RomY complex. We now specifically state that we do not know the oligomeric state of RomY (line 257). Similarly, we do not know the precise stoichiometry of the MglB/RomY, MglA/RomY and MglA/MglB/RomY complexes. However, the interpretation of the overproduction experiments is independent of these stoichiometries. We have added in line 335-339 “These observations corroborate that the rate-limiting step for MglB/RomY GAP activity is complex formation, i.e. when one of the proteins is overproduced in the presence of the other, then more of the MglB/RomY complex is formed, thus, also supporting that the MglB/RomY interaction is low affinity”. And in line 456-460 “Continued biochemical work and structural studies will be required to determine …..the stoichiometry of the MglA-GTP/MglB/RomY complex as well as to decipher the exact mechanism by which RomY binds to the MglA/MglB complex and stimulates MglB GAP activity” to clearly point out that there are still questions to be addressed in future work.

2. Please provide a discussion or a summary table in the supplementary to comment on how these GTPase measurements for MglA compare with the existing published literature that reported the GTPase activities.
**Response:** We added to the main text in line 220-222 that “These specific GTPase activities are similar to those previously reported for MglA-His_6_ alone and MglA-His_6_:His_6_-MglB mixed in a 1:2 ratio (Baranwal et al 2019; Galicia et al 2019; Szadkowski et al 2019)”. For reference, we include below (but not in the manuscript) published specific activities:

|  | Proteins (ratio) | Activity (GTP/MglA/hour) |
| --- | --- | --- |
| Baranwal et al 2019 | MglA | ~1 |
|  | MglA:MglB (1:2) | ~9 |
| Galicia et al 2019 | MglA | ~0.5 |
|  | MglA:MglB (1:2) | ~14 |
| Szadkowski et al 2019 | MglA | ~1.5 |
|  | MglA:MglB (1:2) | ~15 |
| This work | MglA | 2-4 (using 2 different assays) |
|  | MglA:MglB (1:2) | 12-18 (using 2 different assays) |
|  | MglA:MglB:RomY (1:2:2) | 18-31 (using 2 different assays) |

3. It is mentioned that RomY outcompetes RomRX activity. The possibilities of a competition between these two proteins upon interaction with MglAB has not been discussed. The proposed mechanism does not explain how RomY acts as a competitor for RomRX. Hence, the question of restriction of the GEF activity remains unanswered.
**Response:** The epistasis experiments in Fig 1D, E and Fig 4A, B do not support that RomY alone would be able to outcompete RomR/RomX GEF activity. They do also not support that RomY would inhibit RomR/RomX GEF activity. Rather they support that the bipartite MglB/RomY GAP outcompetes RomR/RomX activity at the lagging pole. Similarly, the *in vitro* experiments in Fig 2A and Fig 4C show that RomY alone does not have GAP activity; rather the MglB/RomY complex has GAP activity. Because the idea that RomY alone would outcompete RomR/RomX GEF activity is not supported by any of our observations, we have not pursued this line of experimentation in vitro. In the revised manuscript, we have rewritten the text throughout to clarify the competition model and to emphasize that RomY by almost exclusively localizing at the lagging pole in moving cells contributes to stimulating MglB GAP activity at this pole and is essential for the MglB/RomY GAP activity to outcompete and dominate over the RomR/RomX GEF activity at this pole. We also emphasize that we have no evidence supporting that MglB/RomY interferes with RomR/RomX GEF activity. Similarly, we emphasize that the RomR/RomX GEF complex dominate over the MglB(/RomY) GAP activity at the leading pole because there is little to no RomY at this pole. We extended the Discussion to more clearly bring this idea across. On a separate note, we would also like to add that all our data are in agreement with the competition model. We specifically added in the Discussion (line 478-481) that “In principle, RomY could also help to exclude MglA-GTP from the lagging pole by inhibiting the RomR/RomX GEF at this pole; however, the epistasis experiments (Fig 1D, E) do not support this scenario.” And in line 520-523 “In this model, the RomR/RomX GEF and MglB/RomY GAP complexes are both active at the lagging pole potentially giving rise to futile cycles of GEF and GAP activity with GDP-for-GTP exchange and GTP hydrolysis by MglA. Currently, there is no evidence supporting that the two activities are cross-regulated”.

4. Validation of the AlphaFold model should be discussed.
- One of the possibilities is to use the crosslinking data (used for capturing the transient complexes in pull-down assays) and identify the crosslinked peptides. This also will demonstrate that the captured interactions are indeed specific.
- The AlphaFold model shows that RomY-MglB interface consists mainly the C-terminal helix of MglB. Will a GTPase assay with the deletion construct of C-terminal helix of MglB help in confirming the mechanism of action of the RomY co-GAP and validate the structure proposed?
**Response:** In the revised manuscript, we included in Materials & Methods a description of the quality criteria suggested by AlphaFold. We note that especially for complexes, the pAE is essential to evaluate a structural model. We also switched from using “confidence” to “accuracy” as suggested by AlphaFold. All the models that we discuss are high accuracy models except in the case of RomY beyond residue 90. Consequently, we only discuss how the N-terminal domains of RomY interacts with MglA, MglB2 and the MglA/MglB2 complex. The AlphaFold models suggest that the N-terminal domain of RomY would have a key role in stimulating the GAP activity of MglB. We, therefore, went ahead and tested this prediction in vivo and in vitro. Indeed, we find that this domain has partial RomY activity in vitro (Fig. 2A) and in vivo (Fig. 3C). Altogether (1) the AlphaFold models are high accuracy, (2) they are in agreement with the in vitro data that RomY interacts with MglA and MglB, and (3) they predict that the MglB GAP stimulating activity is in the N-terminal domain. We verify these predictions in vitro. Based on these observations, we feel confident that the AlphaFold model are quite accurate.

Concerning the suggestion to delete the C-terminal α-helix of MglB to validate the AlpfaFold structures: Our understanding is that this helix is important for the interaction between MglA and MglB (Baranwal et al 2019). Therefore, we decided to validate the AlphaFold structure by “targeting” RomY and analyze the N-terminal domain of RomY.

Concerning the specificity of the cross-linking experiments: We observe that RomY can be cross-linked to MglA/GTP but not MglA/GDP as well as to MglB but not MalE. Thus, we have two negative controls in the cross-linking experiments. Given that all the cross-linking experiments are in agreement with the in vivo and in vitro experiments, we feel confident that the cross-linking experiments nicely captures the interactions between RomY and MglA as well as MglB. Concerning the suggestion to identify cross-linked peptides: This is an interesting idea but we strongly feel that this would be well beyond the scope of this manuscript. We write in the Discussion (line 456-460) that “Continued biochemical work and structural studies will be required …….. to decipher the exact mechanism by which RomY binds to the MglA/MglB complex and stimulates MglB GAP activity”.

5. How would you define the concept of a co-GAP? Given that the active site residues are present in MglA itself, and MglB is capable of orienting these, how do you propose that the co-GAP contributes to the increased activity?
**Response:** We realized that co-GAP was probably not a well-chosen term. Therefore, we now refer to RomY as a protein that stimulates MglB GAP activity and that MglB/RomY constitute a bipartite GAP. We do not know the precise mechanism of RomY. We therefore write in the Discussion (line 456-460): “Continued biochemical work and structural studies will be required to …….. decipher the exact mechanism by which RomY binds to the MglA/MglB complex and stimulates MglB GAP activity”.
We would like to add that the precise mechanism of RomY is not essential for the conclusions of the work presented.

6. How do the GTPase activity levels vary with varying ratios of RomY and MglB? As of now, a 1:2 ratio of MglB has been used, and a 1:2 ratio of RomY too. The stoichiometry at which the GTPase activities are saturated will be interesting to see, in order to get estimates of stoichiometry.
**Response:** In the revised manuscript, we included new experiments and measured MglA GTPase activity at different stoichiometries of MglB and RomY. These new experiments, which are included in Fig. 4C and described in line 340-352, demonstrate that with 3µM MglA, GTPase activity slowly saturates from 6µM MglB. This is in agreement with the 1:2 stoichiometry of the MglA:MglB complex. In the titration experiments with Strep-RomY (in the presence of 3µM MglA and 6µM MglB), we find that saturation was reached at 3-6µM Strep-RomY. This observation together with the high accuracy AlpfaFold model with a RomY monomer gives some support to a 1:2:1 stoichiometry of MglA:MglB:RomY complex. Because we only have one line of experimental evidence for this stoichiometry, we still write in the Discussion (line 456-460) “Continued biochemical work and structural studies will be required to determine …… and the stoichiometry of the MglA-GTP/MglB/RomY complex …….”.

7. Has the oligomeric status of RomY been checked? Why was a monomeric state assumed for generating the AlphaFold model? Will alternative models be generated in AlphaFold if RomY was considered as a dimer?
**Response:** We have performed gel filtration experiments on Strep-RomY; however, the data are inconclusive, most likely because a large part of RomY is disordered. In line 257, we write ” Because the oligomeric state of RomY is not known, we modelled RomY as a monomer”. AlphaFold models monomeric RomY with high accuracy. We have also tried to model RomY as a dimer and trimer alone as well as complexed to MglA and/or MglB2. However, all these models are of lower accuracy than those that include monomeric RomY. As mentioned in “6”, the titration experiments gives some support to a 1:2:1 stoichiometry of the MglA:MglB:RomY complex. We would like to point out that whether RomY is a monomer, dimer or trimer does not change any of the conclusions in the manuscript. As mentioned in “6”, we write in the Discussion (line 456-460) “Continued biochemical work and structural studies will be required to determine …… and the stoichiometry of the MglA-GTP/MglB/RomY complex …….”.

Specific questions/clarifications:
1. Are there any estimates of concentration of MglB at the poles, and within the cell? How do the differences in concentration at the two poles correlate with the affinity explanation?
**Response:** To our knowledge, there are no data available on the concentration of MglB in vivo. Based on the many publications on MglB localization, there is probably an estimated 10-fold difference in the amount of MglB at the poles. In the revised text we added a comment in the Discussion (line 524-525): “In future experiments, it will, therefore, be important to determine the overall cellular and polar concentrations of the involved proteins to estimate the magnitude of these futile cycles ……..”.

2. MXAN5749 in UNIProt has been given as 258 aa protein. Explain the discrepancy between the reported sequence in the paper and the database. Since there is a Western blot analysis of the native protein, please provide the protein ladder information so that the size is confirmed.
**Response:** Thanks for pointing this out to us. In the revised text (line 117, 939, 1061), we have now included that we use the annotation from the NCBI Reference Sequence NC_008095.1. (locus tag: MXAN_RS27865). UniProt is still using the old annotation for MXAN_5749. We also note that the size of RomY by SDS-PAGE fits with the new annotation and not the old annotation.

3. Line 192-193: If RomY inhibits RomR/RomX GEF activity, then ΔromY mutation would not suppress the gliding defect in the ΔromR and ΔromX mutants, So it can act as an inhibitor of RomRX GEF, and this inhibition can happen in an MglB dependent manner. So it is not really clear why scenario 1 is excluded.
**Response:** The epistasis experiments in Fig 1D, E do not support that RomY is an inhibitor of RomR/RomX GEF activity. We are also not aware of experimental data suggesting that MglB would inhibit RomR/RomX GEF activity. So, the simplest explanation of the epistasis experiments is that RomY either has GAP activity or stimulates MglB GAP activity. Therefore, we tested the activity of RomY in vitro and found that it stimulates MglB GAP activity. In other words, we have no evidence that RomY and/or MglB directly interferes with RomR/RomX GEF activity. And, therefore, we do not test for that in vitro. We would like to add that all our data are consistent with RomY acting to stimulate the GAP activity of MglB. Nevertheless, we have added in the Discussion (line 523) “Currently, there is no evidence supporting that the two activities are cross-regulated” and (line 478-481) “In principle, RomY could also help to exclude MglA-GTP from the lagging pole by inhibiting the RomR/RomX GEF at this pole; however, the epistasis experiments (Fig 1D, E) do not support this scenario”.

4. Line 211: Why was this assay not done in a 1:2:1 ratio of MglA:MglB:RomY, given that your model proposes a 1:2:1 complex? What happens to the GTPase rate in such a case? Again, is there a further increase in GTPase activity in vitro with increasing concentrations of MglB, beyond 1:2? This experiment is essential to get information on the maximum activity that can be achieved, when MglB is in saturating levels. Similarly, how does concentration variation of RomY affect the activity?
**Response:** We initially chose the 1:2:2 ratio of MglA:MglB:RomY because we had to start somewhere. In the revised manuscript, we have now included new experiments and measured MglA GTPase activity at different stoichiometries of MglB and/or RomY. These new experiments, which are included in Fig. 4C and described in line 340-352, demonstrate that 3µM MglA GTPase activity slowly saturates from 6µM MglB. This is in agreement with the 1:2 stoichiometry of the MglA:MglB complex. In the titration experiments with Strep-RomY (in the presence of 3µM MglA and 6µM MglB), we find that Strep-RomY reaches saturation at 3-6µM. This observation together with the high accuracy AlpfaFold model with a RomY monomer give some support to a 1:2:1 stoichiometry of MglA:MglB:RomY complex. Because we only have one line of experimental evidence for this stoichiometry, we still write in the Discussion (line 456-460) “Continued biochemical work and structural studies will be required to determine …… and the stoichiometry of the MglA-GTP/MglB/RomY complex …….”.

5. Was a pull-down attempted with MglA-GTP-MglB complex without or with the crosslinker? According to the proposed model, an interaction with the MglAB complex might be a more stable interaction, and might be retained even in the absence of a crosslinker.
**Response:** We did indeed try to do the pull-down experiments with and without cross-linking in the presence of all three proteins. However, this experiment is technically challenging to interpret because all three proteins interact in all three pairwise combinations. However, the GTPase assays in the presence of MglB and/or RomY clearly demonstrate that the three proteins can form a complex in which they are all present.

6. Line 204 mentions His6-MglB while 206 mentions MglB-His6. What is the position of the tag? Please correct the text accordingly.
**Response:** We apologize for this confusion. The fusion is His_6_-MglB and we have corrected the text throughout.

7. RomY could increase the affinity of MglB homo-dimer for MglA. This statement implies that an increased concentration of MglB should result in an increase of GTPase activity independent of RomY addition. The GTPase activity measurements with increased MglB concentrations (increasing ratios till it reaches saturation) will help in coming up with a mechanism of co-GAP. An explanation of how this does not happen within the cell should be provided.
**Response:** In the revised manuscript, we included new experiments and measured MglA GTPase activity at different stoichiometries of MglB and RomY. These new experiments, which are included in Fig. 4C and described in line 340-352, demonstrate that with 3µM MglA, GTPase activity slowly saturates from 6µM MglB. This is in agreement with the 1:2 stoichiometry of the MglA:MglB complex. In the titration experiments with Strep-RomY (in the presence of 3µM MglA and 6µM MglB), we find that saturation was reached at 3-6µM Strep-RomY. This observation together with the high accuracy AlpfaFold model with a RomY monomer gives some support to a 1:2:1 stoichiometry of MglA:MglB:RomY complex. Because we only have one line of experimental evidence for this stoichiometry, we still write in the Discussion (line 456-460) “Continued biochemical work and structural studies will be required to determine …… the stoichiometry of the MglA-GTP/MglB/RomY complex as well as to decipher the exact mechanism by which RomY binds to the MglA/MglB complex and stimulates MglB GAP activity”.

8. Line 278 - 280: MglA and MglB form a tight complex, which can be retained in SEC experiments and with KD of about 1 uM. This also results in a stimulation of MglA GTPase activity, and efficiently orients the catalytic residues. Mechanistically, the requirements of a coGAP is not clear. MglA active site is well formed with just the interaction with MglB. Hence, the role of RomY could be to further stabilise the MglA/MglB interaction? Since there is no requirement for a supply of active site residue for the GTPase from the GAP or co-GAP, the function of the co-GAP should be stabilization of the active site conformation, a role proven to be performed by MglB alone. In this context, how can the requirement of RomY be explained? Having a approximate estimates of protein concentrations at the poles during reversals will be interesting information, if available.
**Response:** To our knowledge, there are no data available on the concentration of MglB in vivo. Based on the many publications on MglB localization, there is probably an estimated 10-fold difference in the amount of MglB at the poles. In the revised text we added a comment in the Discussion (line 524-525): “In future experiments, it will, therefore, be important to determine the overall cellular and polar concentrations of the involved proteins to estimate the magnitude of these futile cycles ……..”. Similarly, we do not know the mechanism of RomY. Therefore, we write (line 456-460) “Continued biochemical work and structural studies will be required to ….. decipher the exact mechanism by which RomY binds to the MglA/MglB complex and stimulates MglB GAP activity”. We would like to add that the MglA/MglB structure was obtained by X-ray crystallography, which gives a single snapshot of the active site but does not allow to catch the dynamics of the active site. Therefore, it is a possibility that RomY could stabilize the active site in MglA.

9. Line 309: It is not entirely clear why an overproduction of MglB in the absence of RomY will not affect reversal frequencies? We do observe a dose dependent activation of the MglA GTPase activity by MglB (Zhang et al 2010, Fig 1C). Kindly clarify why it doesn't have an effect in-vivo. Considering the mechanism of activation by a co-GAP based on stabilisation of interaction between MglA and MglB, it seems surprising that this effect is not observed.
**Response:** In the new experiments in Fig. 4C and line 340-352, we also see a concentration dependent activation of MglA by MglB. However, it is important to emphasize that the conditions in vivo and in vitro are very different. To clarify, we included in line 358-369 “….several factors make a direct comparison between the *in vitro* and *in vivo* data difficult. First, in the *in vitro* system, GAP activity is measured in the absence of GEF activity; however, *in vivo*, both activities are present. Therefore, overproduced MglB *in vivo* may have GAP activity, but it is too low to outcompete the GEF activity of the RomR/RomX complex (see Discussion). Second, the cellular and polar concentrations of MglA, MglB and RomY are not known and, therefore, it is not known where on the titration curves the relevant physiologically concentrations are. Third, His_6_-MglB alone at very high concentrations *in vitro* stimulated MglA-His_6_ GTPase activity almost to the same level as His_6_-MglB together with Strep-RomY. Nevertheless, the ~20-fold overproduction of MglB in the absence of RomY was not sufficient to reach the MglB/RomY GAP activity in WT. Thus, it remains a possibility that if MglB could be overproduced to even higher levels, then MglB alone would be sufficient to display detectable GAP activity *in vivo*”.

10. Line 327: “In the absence of RomR, RomY-YFP polar localization was also decreased”. Elaborate on the role of RomR on RomY localisation. Why do you observe such a phenotype? This has not been discussed further.
Line 327: Is there a decrease in RomY levels in the absence of RomR (the band in Figure S7 looks less compared to the others). Is this significant over multiple repeats? Can this be a cause for the decrease in polar localization?
**Response:** We apologize for this confusion. In the revised text in line 386-393, we now clarify that “In the absence of RomR, RomY-YFP polar localization was also strongly reduced, and only 54% of cells still had a weak polar signal (Fig. 5A). Of note, polar localization of MglB, MglA and RomX is strongly reduced or even abolished in the absence of RomR [29, 33, 35, 36], but MglA, RomR and RomX are still polarly localized in the absence of MglB [36]. In agreement with the direct interaction between RomY and MglB, we, therefore, conclude that MglB is the primary polar targeting determinant of RomY-YFP, and that the reduced polar localization of RomY-YFP in the absence of RomR is caused by an indirect effect of RomR on MglB localization”.

Concerning the decrease in RomY-YFP in the absence of RomR in the western in Fig. S7B: We repeated this western and did not observe a decrease in RomY-YFP. We apologize for the confusion and have included the new western in Fig. S7.

11. RomY native levels with overexpression of MglB leads to low MglA-GTP levels: How will this compensate for the increased requirement of 1 RomY per MglAB complex formed (according to the proposed AlphaFold model)? Please clarify this in the text.
**Response:** Because overproduction of MglB (in the presence of WT levels of RomY) and overproduction of RomY (in the presence of WT levels of MglB) results in increased GAP activity, we infer that in WT cells, RomY is not saturated by MglB and MglB is not saturated by RomY. MglB and RomY interact to form a complex and follow the general reaction scheme MglB2 + RomY ⇌ MglB2/RomY. Under steady state conditions, the ratio between MglB2 and RomY vs MglB2/RomY is constant. Thus, if MglB or RomY are overproduced in otherwise WT cells, this reaction is “pushed” towards formation of the MglB2/RomY complex. In other words, more of the MglB2/RomY complex will be generated independently of the precise stoichiometry of the MglB/RomY complex when MglB or RomY is overproduced. Consequently, the cellular level of the active MglB2/RomY GAP increases upon MglB or RomY overproduction. To make this point more clear we included in line 335-339 “These observations corroborate that the rate-limiting step for MglB/RomY GAP activity is complex formation, i.e. when one of the proteins is overproduced in the presence of the other, then more of the MglB/RomY complex is formed, thus, also supporting that the MglB/RomY interaction is low affinity”.

12. Line 343: “in the absence of RomY, polar localization of MglA-mVenus was increased and switched toward more bipolar symmetric”. Does this observation actually provide proof for showing that inhibition of RomRX at the lagging pole was lifted hence RomRX can recruit MglA-GTP to both the poles, validating Scenario-1 in line 193?
**Response:** In all the in vivo experiments with the Δ*romY* mutant, it phenocopies the Δ*mglB* mutant. Moreover, the epistasis experiments in Fig. 1DE strongly support that RomY has the same function as MglB and that RomY does not interfere with RomR/RomX activity, i.e. the epistasis experiments do not support scenario 1. Along these lines, we find in vitro that RomY stimulates MglB GAP activity. In agreement, we find in the cell biology experiments in which we determine the localization of MglA-mVenus in the absence of RomY that MglA-mVenus localizes in a more bipolar symmetric pattern in the absence of RomY. The same is observed in the absence of MglB. So, the localization of MglA in the absence of RomY also supports that RomY is essential for stimulating MglB GAP activity to a sufficient level to be able to regulate polarity. We added to the Discussion in line 478-481 “In principle, RomY could also help to exclude MglA-GTP from the lagging pole by inhibiting the RomR/RomX GEF at this pole; however, the epistasis experiments (Fig 1D, E) do not support this scenario”.

13. Lines 359-361: Most of the data points for MglB-mVenus seems to be at a unipolar localization? Why is it given that MglB-mVenus is bipolar asymmetric?
**Response:** We apologize for the confusion with this figure. We redid the figure to clarify. Importantly, the data in this figure clearly demonstrates that RomY is almost exclusively at the lagging pole while MglB is clearly bipolar asymmetric.

14. Line 394: Even when MglB was overproduced, its activity was dependent on RomY. This does not corroborate with just a requirement for stabilization of the complex. Given the stoichiometry proposed in the model, a 20-fold overproduction of MglB should in principle help in attaining the highest turnover number for MglA, and compensate for any decrease in GTPase activity due to dissociation of the MglA/MglB complex.
**Response:** To clarify, we included in line 358-369 “….several factors make a direct comparison between the *in vitro* and *in vivo* data difficult. First, in the *in vitro* system, GAP activity is measured in the absence of GEF activity; however, *in vivo*, both activities are present. Therefore, overproduced MglB *in vivo* may have GAP activity, but it is too low to outcompete the GEF activity of the RomR/RomX complex (see Discussion). Second, the cellular and polar concentrations of MglA, MglB and RomY are not known and, therefore, it is not known where on the titration curves the relevant physiologically concentrations are. Third, His_6_-MglB alone at very high concentrations *in vitro* stimulated MglA-His_6_ GTPase activity almost to the same level as His_6_-MglB together with Strep-RomY. Nevertheless, the ~20-fold overproduction of MglB in the absence of RomY was not sufficient to reach the MglB/RomY GAP activity in WT. Thus, it remains a possibility that if MglB could be overproduced to even higher levels, then MglB alone would be sufficient to display detectable GAP activity *in vivo*”.

15. Line 404 - 406: ‘formation of the complex rather than protein concentrations’ is an ambiguous statement, any complex formation, irrespective of the affinities is dependent on protein concentrations. I wonder if the authors imply stoichiometries here? This sentence has been included in various instances in the manuscript and might need to be rephrased.
**Response:** We apologize for this confusion and have deleted this statement throughout.

16. If the MglA-GTP-RomY interaction occurs as proposed in the AlphaFold model, RomY should be able to stabilise the binding of GTP to MglA. Has this been tested experimentally?
**Response:** No this has not been tested experimentally. The question of the reviewer probably comes back to the mechanism of RomY and how precisely RomY stimulates MglB GAP activity. This mechanistic aspect will be addressed in future work (see line 456-460).

17. Line 417: “this localization pattern is ideal to allow stable and switchable polarity”. It is still not clear why the GEF complex would accumulate majorly at the lagging pole only to be outcompeted by the GAP complex? This is very unlike what is usually seen as per the localised activity of the eukaryotic GEF-GTPase.
**Response:** We have added a brief explanatory sentence (line 496-497) to clarify why it is ideal for cells to have the RomR/RomX GEF activity at the lagging pole. In addition, we have included ref 36 in which the trade-off is explained in details.

Minor points:
1. Fig 1E. 4th lane (delMglA) has not been marked as NA.
**Response:** Thanks & done!

2. Line 91: MglA GDP is diffused in the cytoplasm
**Response:** Thanks & done!

3. Line 182: How can it be concluded that RomY acts downstream of the Frz system? Though I understand that it is related to MglAB and RomXY, without epistasis data involving Frz genes, the claim in this sentence cannot be made. This should be mentioned as a hypothesis.
**Response:** These epistasis experiments are included in Fig. S2. In the revised manuscript, we are now more clearly referring to this figure (line 157 and 186-191).

4. Lines 241-245: AlphaFold predictions for existing structures are usually very reliable because the structural information is used in generating the prediction. In general, AlphaFold also tends to pick up conformations which are more represented in the PDB. Hence, it is not accurate to claim that RomY interacts with the GTP-bound conformation based on the AlphaFold model.
**Response:** The AlphaFold structural model for the MglA/MglB2 complex is in agreement with the solved structure of the complex. Similarly, the AlphaFold structural models catch MglA in the GTP-bound form when complexed with RomY and with MglB2/RomY. Based on these models, we write (line 286-289) “….the MglA:(MglB)_2_:RomY model *supports* that all three proteins interact to form a complex in which they interact in all three pairwise directions and in which MglA is in the GTP-bound state. Also, this model agrees with the interactions detected in the pull-down experiments (Fig 2B; S3C, S3D Fig)”. In other words, we do not conclude that RomY interacts with the MglA-GTP bound form. We write that the AlphaFold models support that RomY interacts with MglA-GTP. And, that this is in agreement with the in vitro data.

5. Line 267: “RomYN stimulated MglA GTPase activity in the presence of MglB almost as efficiently as full-length RomY”. However, it appears from Fig 2A, that the difference between the activities of RomYN and RomY full length might be significant?
**Response:** Thanks & rewritten to “Strep-RomY^N^ stimulated MglA GTPase activity in the presence of MglB, although at a slightly but significantly lower level than full-length Strep-RomY” (line 294).

6. Line 301: “We conclude that MglB and RomY accumulate independently of each other “ This might be confused with MglB dependent localisation of RomY, and hence should be rephrased to remove ambiguity.
**Response:** We tried to clarify this sentence (line 328).
